# Design and preclinical testing of an anti‐CD41 CAR T cell for the treatment of acute megakaryoblastic leukaemia

**DOI:** 10.1111/jcmm.17810

**Published:** 2023-09-04

**Authors:** Adrian Bogdan Tigu, Catalin Sorin Constantinescu, Patric Teodorescu, David Kegyes, Raluca Munteanu, Richard Feder, Mareike Peters, Ioana Pralea, Cristina Iuga, Diana Cenariu, Andra Marcu, Alina Tanase, Anca Colita, Rares Drula, Jon Thor Bergthorsson, Victor Greiff, Delia Dima, Cristina Selicean, Ioana Rus, Mihnea Zdrenghea, Diana Gulei, Gabriel Ghiaur, Ciprian Tomuleasa

**Affiliations:** ^1^ Medfuture Research Center for Advanced Medicine Iuliu Hatieganu University of Medicine and Pharmacy Cluj‐Napoca Romania; ^2^ Department of Hematology Iuliu Hatieganu University of Medicine and Pharmacy Cluj‐Napoca Romania; ^3^ Intensive Care Unit Emergency Clinical Hospital Cluj‐Napoca Romania; ^4^ Department of Leukemia, Sidney Kimmel Cancer Center at Johns Hopkins Johns Hopkins University School of Medicine Baltimore Maryland USA; ^5^ Department of Drug Analysis School of Pharmacy Iuliu Hatieganu University of Medicine and Pharmacy Cluj‐Napoca Romania; ^6^ Department of Pediatrics Carol Davila University of Medicine and Pharmacy Bucharest Romania; ^7^ Department of Stem Cell Transplantation Fundeni Clinical Institute Bucharest Romania; ^8^ Stem Cell Research Unit, Biomedical Center, School of Health Sciences University of Iceland Reykjavík Iceland; ^9^ Department of Laboratory Hematology Landspitali University Hospital Reykjavík Iceland; ^10^ Department of Immunology University of Oslo and Oslo University Hospital Oslo Norway; ^11^ Department of Hematology Ion Chiricuta Clinical Cancer Center Cluj Napoca Romania

**Keywords:** B cell, CAR T cells, CRS, megakaryoblastic leukaemia

## Abstract

Acute megakaryoblastic leukaemia (AMkL) is a rare subtype of acute myeloid leukaemia (AML) representing 5% of all reported cases, and frequently diagnosed in children with Down syndrome. Patients diagnosed with AMkL have low overall survival and have poor outcome to treatment, thus novel therapies such as CAR T cell therapy could represent an alternative in treating AMkL. We investigated the effect of a new CAR T cell which targets CD41, a specific surface antigen for M7‐AMkL, against an in vitro model for AMkL, DAMI Luc2 cell line. The performed flow cytometry evaluation highlighted a percentage of 93.8% CAR T cells eGFP‐positive and a limited acute effect on lowering the target cell population. However, the interaction between effector and target (E:T) cells, at a low ratio, lowered the cell membrane integrity, and reduced the M7‐AMkL cell population after 24 h of co‐culture, while the cytotoxic effect was not significant in groups with higher E:T ratio. Our findings suggest that the anti‐CD41 CAR T cells are efficient for a limited time spawn and the cytotoxic effect is visible in all experimental groups with low E:T ratio.

## 1 INTRODUCTION

Before the publishing of the international consensus classification of myeloid malignancies and acute leukaemias,^1^ Acute megakaryoblastic leukaemia (AMkL) was formerly classified as M7 subtype (M7‐AML) and represents no more than 5% of all acute myeloid leukaemias (AML). AMkL is characterized by a proliferation of more than 20% megakaryoblasts which are identified by flow cytometry based on their specific antigens.^2^, ^3^ This disease is a frequent AML diagnosed in children with a high frequency in children with Down syndrome,^4^, ^5^, ^6^, ^7^ while in adult, M7‐AML accounts 1% of the total diagnosed cases. The disease was first mentioned in 1931 and since 1985 is classified by FAB (French‐American‐British classification of AML); however, until now the clinical experience is limited, with few published data regarding the chromosomal abnormalities.^2^, ^8^, ^9^ The patients diagnosed with AMkL have poor response to treatment and low overall survival.^10^, ^11^

In the last decades, significant progress has been made in the field of novel immunotherapeutic agents that can be used as treatments for haematological malignancies. Chimeric antigen receptor T cells (CAR T cell) are the T cells modified to recognize specific antigen.^12^, ^13^, ^14^ The structure of a CAR T cell that is currently used in clinics includes scFv—the single‐chain variable domain of an antibody which is responsible for the antigen specificity, a transmembrane domain, TCR—the signal transduction domain of T‐cell receptor which can allow the intracellular activation, and one or more intracellular costimulatory domains.^15^, ^16^, ^17^ Currently, five FDA‐approved (Food and Drug Administration) CAR‐T cell therapies are used in the clinic. This is the case for the anti‐CD19 CAR T cells tisagenlecleucel,^18^ brexucabtagene autoleucel,^19^ lisocabtagene maraleucel and axicabtagene ciloleucel,^20^ as well as the anti‐BCMA idecabtagene vicleucel.^21^ Thus, the existing CAR T cells are mostly targeting CD19, which is a specific antigen for B‐cells, with impressive results in B‐cell acute lymphoblastic leukaemia and B‐cell non‐Hodgkin lymphomas.^22^

As AMkL, despite being a rare subtype of AML, the purpose of the current manuscript is to design and afterwards investigate in the preclinical setting an anti‐CD41 CAR T cell, both in vitro and in vivo.

## 2 MATERIALS AND METHODS

### 2.1 Design of the CAR construct

**FIGURE 1:**
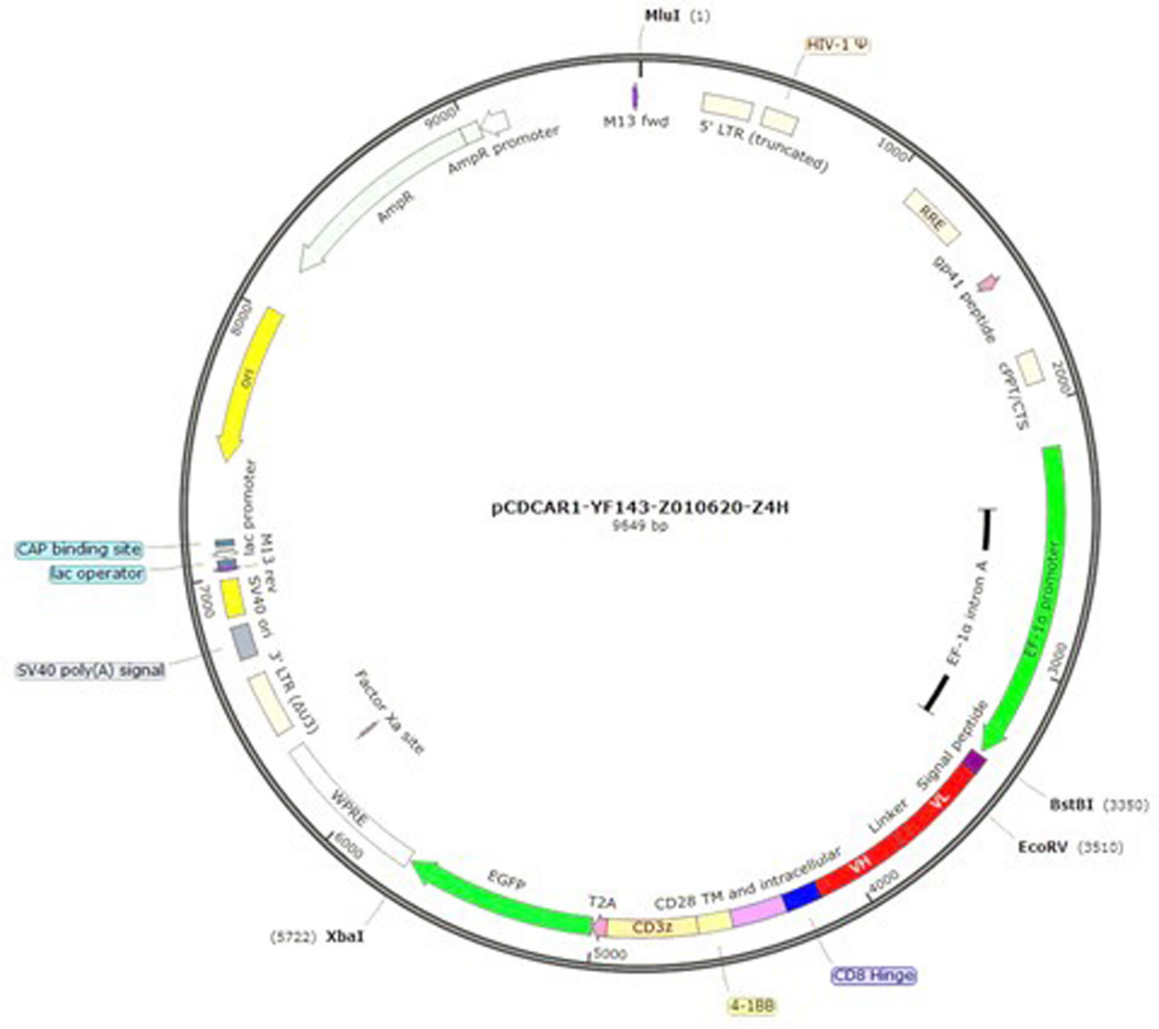
Scheme of CAR construct of pCDH‐EF1a‐scFv (Abciximab)‐28BBζ‐GFP‐CAR.

The full length of chimeric antigen receptor was synthesized and subcloned into lentivirus vector by Creative Biolabs (Creative Biolabs, Shirley, NY, USA). The insert was afterwards confirmed by Sanger sequencing (Figure 1).

### 2.2 Transfection of the T lymphocytes and obtaining CAR T cells

**FIGURE 2:**
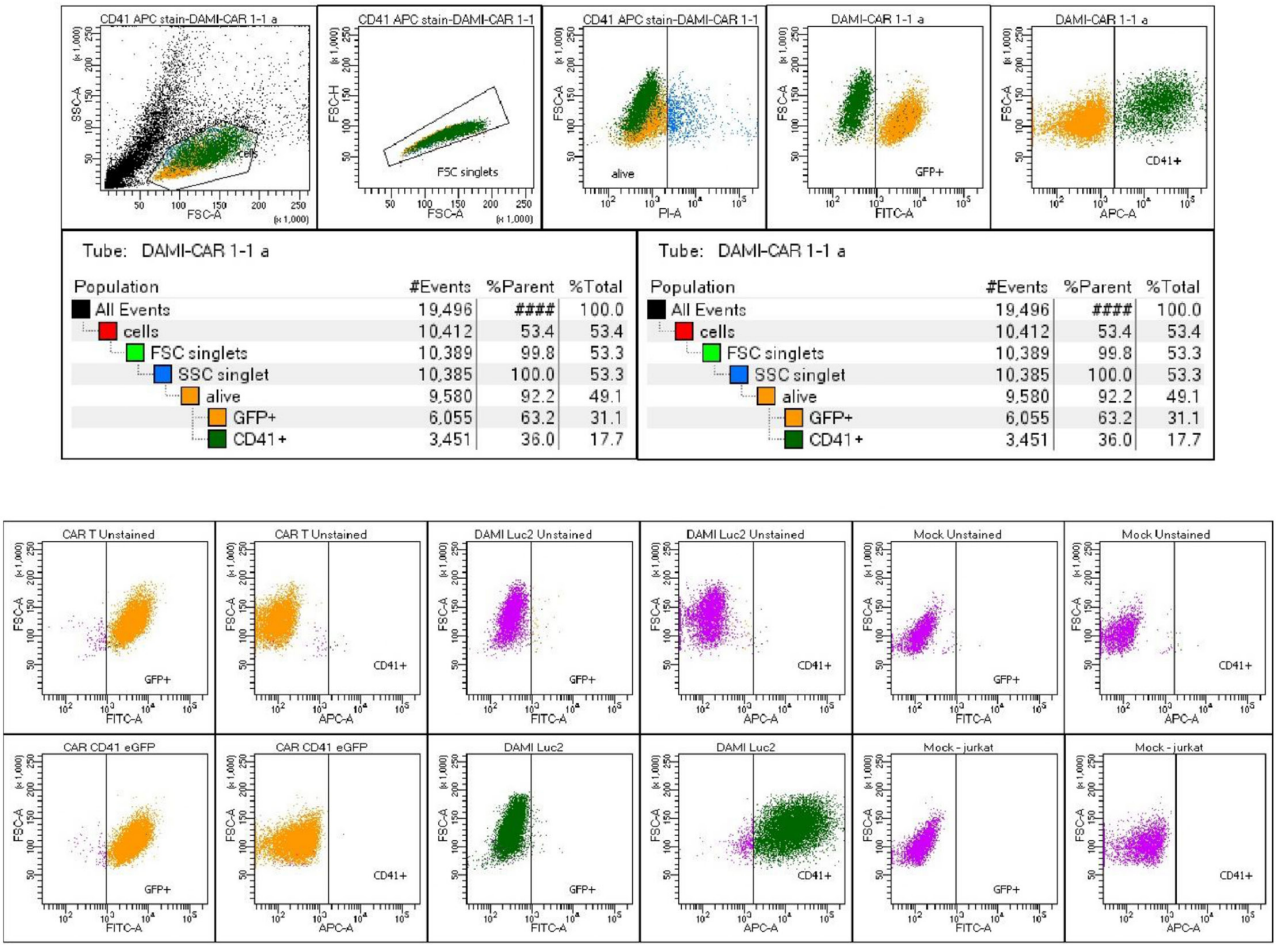
Gating strategy for CAR T cells, DAMI Luc2 cells and Mock (Jurkat cells) after P.I. selection of alive cells. The top left represents the Cells selection out of all events recorded. The second gate represents the singlets which will be further analysed based on the P.I signal in the third gate. The negative P.I. negative cells are further gated based on the FITC signal (fourth gate) and APC signal (Fifth gate). The GFP‐positive and CD41‐positive cells were analysed from the alive cells. The cell populations were first analysed unstained to select the negative populations for CD41‐APC. Only CAR T cells show a positive signal at unstained populations, only for eGFP signal from the eGFP that is inserted in the cell membrane with the CAR construct. DAMI cells are positive for CD41‐APC, while CAR T cells and Mock cells are negative for CD41‐APC.

2 × 10^5^ Jurkat cells were transduced with the viral vector through spinoculation. Cells were incubated with 10% viral vector and Polybrene at a concentration of 8 ug/mL, and centrifuged for 90 min, at 1300 *g*, 37°C. Afterwards, the cells were expanded in culture, and GFP‐positive cells were FACS‐sorted. After further expansion, the final population was 93.8% GFP‐positive CAR T cells (Figure 2).

### 2.3 Cell culture and reagents

DAMI (CRL‐9792) and Jurkat cells (TIB‐152) were purchased from the American Type Culture Collection (ATCC). Jurkat cells were further processed, and the CAR construct was inserted in the cell genome, further the cell line containing the construct being named CAR Jurkat GFP+. DAMI cells were transfected with the luciferase vector and thus luciferase‐positive cells were obtained (DAMI‐Luc). Both cell lines were maintained in RPMI medium supplemented with 10% fetal bovine serum, 1% glutamine and 1% penicillin–streptomycin. The culture medium and all the supplements were purchased from Gibco. During the experiments, all the cells were maintained in a sterile and humidified chamber with 5% CO_2_.

### 2.4 Co‐culture experiments

To investigate the inhibitory effect of CAR Jurkat GFP+ cells which target CD41 antigen on the surface of DAMI Luc2, seven experimental groups were seeded in triplicate in T25 flasks, starting with a total of 1 million cells/flask in 5 mL final volume. The experimental groups include: DAMI Luc2 alone, CAR Jurkat GFP+ alone, DAMI Luc2: CAR Jurkat GFP+ at 1:1, 1:4, 1:9, 4:1 and 9:1 ratio. Thus, different combinations were evaluated at three time points: 24, 48 and 72 h.

CAR T cells culture media was supplemented with IL‐2 (10 ng/mL; Gibco) for 7 days to activate the T cells. The CAR T cells were further used for co‐culture experiments. For the evaluation of unspecific binding of CAR T cells, 24 h co‐cultures were established with Mock Jurkat cells and CAR T cells with target cells. The populations were evaluated by flowcytometry. The results are presented in Table S1.

### 2.5 Co‐culture evaluation by flow cytometry analysis

DAMI Luc2 and CAR Jurkat GFP+ cells were cultivated as previously mentioned. The experimental groups included both single cell type culture and co‐culture at different ratios between DAMI Luc2 (the target) and CAR Jurkat GFP+ cells (the effector). The cells were cultivated at a starting number of 1 million cells/T25 flask, according to the different ratios for each co‐culture group. After 24, 48 and 72 h, the cells from all experimental groups were washed three times with phosphate buffer saline 1X (PBS1X; Gibco) by centrifugation at 370 × *g*, for 5 min at room temperature. Then, each cell pellet obtained after the washing steps was resuspended in 50 μL of PBS1X containing allophycocyanin ‐ anti CD41 antibody (2 μL/sample; BD) and incubated in dark for 15 min. This step was followed by three washing steps with PBS1X and then incubated with 500 μL of PBS1X containing propidium iodide (P.I; BD) and incubated for 5 min in dark. Following this step, the samples were analysed using the FACS BD CANTO II Flow cytometer (BD).

The gating strategy was based on the allophycocyanin (APC) signal using APC channel, the green fluorescent protein (eGFP) signal using FITC channel and the P.I signal using the phycoerythrin (PE) channel. First, using the SSC‐A and FSC‐A the total cell population was gated and selected, from the total cell population, the singlets were selected by the FSC‐W and FSC‐A, then the alive cells were selected using the PE channel, all events bellow 10^3^ were considered alive. Further, from the alive events, using eGFP signal and CD41‐APC‐A signal, DAMI cells were selected as CD41 positive and eGFP negative, while the CAR Jurkat GFP+ cells were selected as positive for eGFP and negative for CD41‐APC‐A. Each cell line was tested for eGFP and CD41‐APC‐A before starting the experiment to generate a precise gating for positive and negative populations.

### 2.6 LDH Assay

The LDH activity was evaluated with the PicoProbe LDH Cytotoxicity Fluorometric Assay Kit (BioVision). The LDH was measured using 10 μL of the cell supernatant to detect the LDH released from the cells after membrane damage, mixed with the buffer solution and PicoProbe up to 200 μL as in the manufacturer's protocol. The mixture was incubated in a black fluorescence 96 well plate and read at λEx/Em = 535/587 nm. The fluorescence intensity indicates the intensity of the LDH activity. The Fluorometric measurements were performed using CLARIOstar spectrophotometer (BMG Labtech). The results are expressed as mean values and the experimental groups were compared to the control group, DAMI Luc2 cells without CAR T cells in co‐culture.

### 2.7 DAMI Luc2 evaluation by EUROFLOW panels

For a better inclusion of our DAMI Luc2 cell line in the M7‐AMkL subgroup, the cell line samples were analysed using EUROFLOW panels for AMkL (M7) and Erythroid leukaemia (M6) applying the common clinical laboratory diagnostic protocol as for patient samples. The DAMI Luc2 cells were included as M7‐AMkL positive sample and treated as M7‐AMkL cells for this study. The results are described in Figure S1.

### 2.8 Luciferase signal evaluation by IVIS

Co‐cultures of target cells with CAR T cells and Mock were established in a 96 well plate, starting from a total of 10,000 cells per well. The experimental groups (in duplicate) were incubated for 24 h and afterwards 3 μL RediJect D‐Luciferin (XenoLight, Perkin Elmer) were added to each 200 μL of culture media. The plate was then incubated for 10 min and read by IVIS SPECTRUM–IVIS Imaging System (Perkin Elmer) via the bioluminescent reporter optimized for imaging–RediJect D‐Luciferin (XenoLight, Perkin Elmer).

### 2.9 ELISA for the T cell secreted TNF alpha

BioVendor Human TNF alpha ELISA Kit (BioVendor) was used to determine the concentration of TNF alpha in culture media of DAMI Luc2 cells co‐cultured with Mock and CAR T cells for 24 h. The assay was conducted according to the manufacturer protocol. The standard curve was plated together with the samples representing each co‐cultured group (in duplicates). The sample absorption at 450 nm was evaluated by TECAN SPARK 10M (TECAN). The results were expressed in ng/mL of TNF alpha.

### 2.10 Preclinical murine testing of CAR‐T cells against M7‐AML Luc2 positive cells and IVIS evaluation of M7‐AML Luc2 signal

The effect of CAR T cells in M7‐AML was evaluated in M7‐AML animal model, using 8 weeks old NSG‐S female mice, purchased from Charles River Laboratories, lnc. The mice were divided into four groups: control group inoculated with 2.5 million DAMI Luc2 cells, one group inoculated DAMI Luc2 cells and 2 million CAR T cells and one group inoculated with DAMI Luc2 cells and 10 million CAR T cells. First, DAMI Luc2 cells were injected in mice femoral head. After 20 days the CAR T cells were intraperitoneal (i.p) injected.

The tumour growth was evaluated macroscopically and each day, at day 0, after 24 and 48 h the tumour growth was evaluated by IVIS Spectrum – IVIS Imaging System (Perkin Elmer) via the bioluminescence reporter optimized for in vivo imaging – RediJect D‐Luciferin (XenoLight, Perkin Elmer). Due to the aggressiveness of DAMI Luc2 cells and the side effects caused by the CAR T cells inoculation our preliminary study was limited to 48 h after CAR T cells treatment. Bioluminescent images were processed using Living Image® 4.5.2 Software, the software was used to automatically measure the signal intensity within the region of interest using automatic contour tool (ROI). The total counts were further used to compare the intensity between the groups using GraphPad 8 software.

At the end of the experiment the mice were euthanized by anaesthetic overdose.

All experimental protocols were approved by the Ethical Committee of Iuliu Hatieganu University of Medicine and Pharmacy and the experiments were conducted according to the EU Directive 63/2010 and in compliance with the Project Approval no. 237 from 22 December 2020.

### 2.11 Statistical analysis

For the flow cytometry population analysis, the experimental groups were analysed using the two‐tailed paired *t*‐test by comparing the mean value for the control groups with theoretical values from the starting of the experiment and the experimental groups with *p* < 0.05*, *p* < 0.01** and *p* < 0.001***. For the LDH assay, the experimental groups were compared to DAMI group that was not co‐cultured with the CAR T cells, the results after 24 h of incubation were analysed using the two‐tailed paired *t*‐test, with *p* < 0.05*, *p* < 0.01** and *p* < 0.001***. For all the analysed groups, *n* = 3. For the TNF alpha ELISA, the results after 24 h of incubation were analysed using the two‐tailed paired *t*‐test, with *p* < 0.05*, and *n* = 2.

## 3 RESULTS

### 3.1 Anti‐CD41 construct

The anti‐CD41 CAR construct ‐ pCDH‐EF1a‐scFv (Abciximab)‐28BBζ‐GFP‐CAR is shown in Figure 1. The scFv sequence is depicted in Table S2. The nucleotide sequence of the CAR cassette (EF‐1alpha promoter‐Signal peptide‐scFv‐CD8 hinge‐CD28 TM and ICD‐4‐1BB‐CD3ζ‐T2A‐GFP) is shown in Table S3. The aminoacid sequence of the CAR cassette (Signal peptide‐scFv‐CD8 hinge‐CD28 TM and ICD‐4‐1BB‐CD3ζ‐T2A‐iCas9) is shown in Table S4.

### 3.2 Anti‐CD41 CAR T cell

Before starting the experiments, the anti‐CD41 CAR T cells were tested for their GFP signal, to evaluate the percentage of GFP‐positive population. Thus, 1 × 10^5^ CAR T cells were stained with P.I. to eliminate the dead cells and the alive cells were analysed for the eGFP signal. All the events above 10^3^ for eGFP signal were considered GFP‐positive. At the beginning of our experiment, 93.8% of CAR T cells were positive for GFP, indicating that these percentage represent the cells that have the CAR construct inserted into the genome (Figure 2).

### 3.3 Co‐culture ratio optimisation for the inhibition of AMkL cells

Regardless of recent improvement in the therapeutic outcome of AMkL, many cases relapse or are refractory to treatment.^23^, ^24^ Five different co‐culture ratios between the target and effector cells were established. DAMI Luc2 cells, expressing CD41, were used as model for AMkL, while our newly developed CAR T cells which targets CD41 were used as effectors. The co‐culture was evaluated at 24, 48 and 72 h, and the results were compared to the initial seeded populations. The evaluation of the cells populations was performed by flow cytometry, where the GFP+ cells were the CAR T cells which have GFP on their membrane, while DAMI Luc2+ cells are negative for GFP signal. The GFP events were compared to CD41‐APC+ events assigned to DAMI Luc2+ cells, while CAR T cells are negative for APC signal. The alive cells were selected by P.I signal, all the events positive for PI were excluded being considered not alive.

**FIGURE 3:**
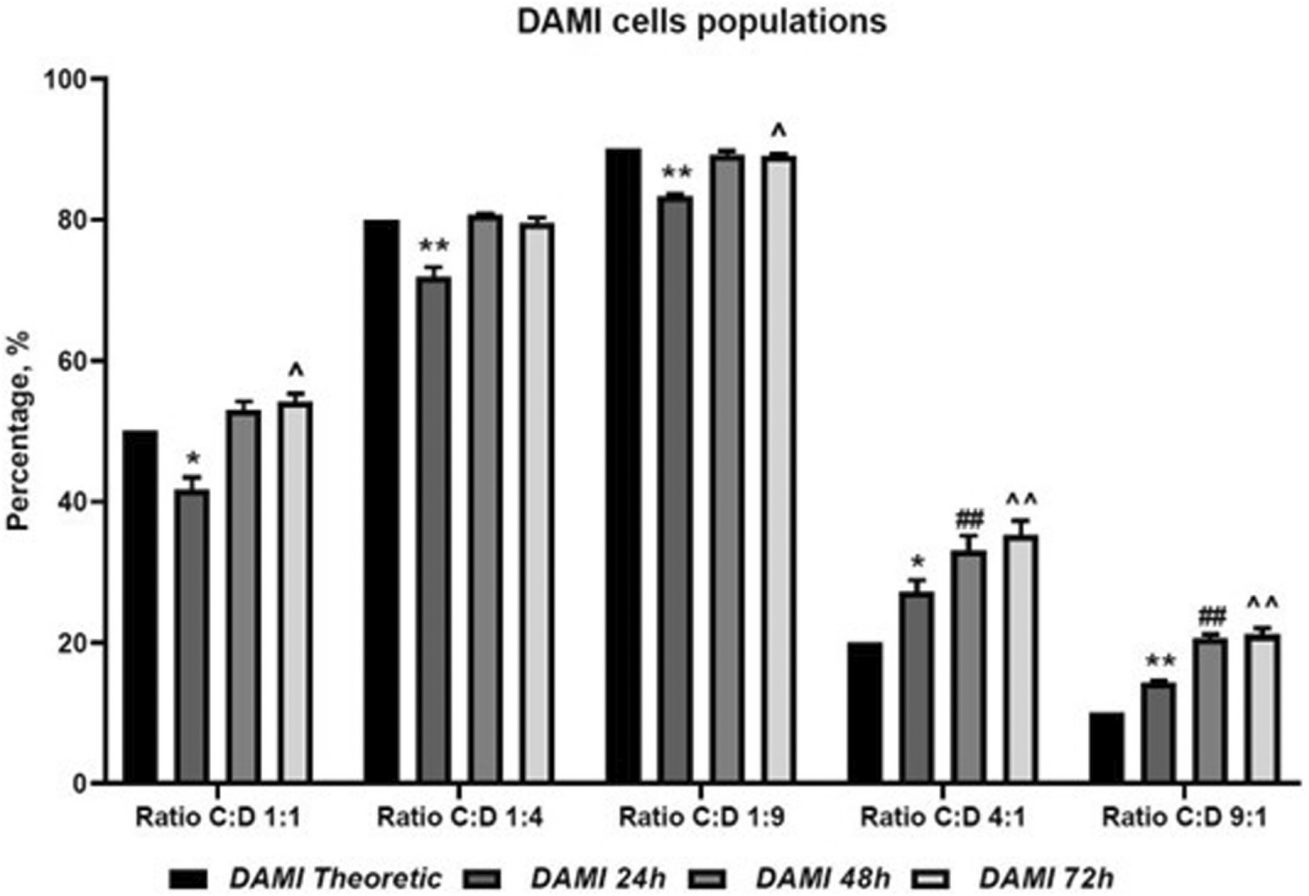
DAMI Luc2 cells in co‐culture after 24, 48 and 72 h. Comparison with the theoretical ratio for each group. the experimental groups were analysed using the two‐tailed paired t‐test by comparing the mean value for the control groups with theoretical values from the starting of the experiment and the experimental groups with *p* < 0.05*, *p* < 0.01** and *p* < 0.001*** when comparisons were made with the DAMI theoretic group. For all the analysed groups, *n* = 3. Legend: * ‐ for comparison of 24 h versus theoretic; # ‐ for comparison of 48 h versus theoretic; ^ ‐ for comparison of 72 h versus theoretic; C:D, CAR T cells versus DAMI cells, at different culture ratio; DAMI – DAMI Luc2+ cells at different time points; ns – not significant.

The co‐culture evaluation shows that CAR T cells have limited inhibitory effect on DAMI Luc2, with the highest inhibitory effect observed at 24 h of co‐culture, in the groups with 50%, 20% and 10% CAR T cells. In the groups with more CAR T cells, we observed no inhibition in DAMI Luc2+ cells and we assume that the increased number of CAR T cells that were initially seeded created clusters (an effect that was observed in culture plates) and less CAR T cells were available for binding DAMI Luc2+ cells (Figure 3, Table 1).

**TABLE 1:** The experimental groups were analysed using the two‐tailed paired *t*‐test by comparing the mean value for the control groups with theoretical values from the starting of the experiment and the experimental groups with *p* < 0.05*, *p* < 0.01** and *p* < 0.001*** when comparisons were made with the DAMI theoretic group.

Group	T versus 24 h	T versus 48 h	T versus 72 h
1:1	0.0138 *	0.0507 ns	0.0274 ^
1:4	0.0097 **	0.0591 ns	0.3715 ns
1:9	0.0011 **	0.1201 ns	0.0225 ^
4:1	0.0158 *	0.0085 ##	0.0060 ^^
9:1	0.0023 **	0.0012 ##	0.0025 ^^

*Note*: For all the analysed groups, *n* = 3. * ‐ for comparison of 24 h versus theoretic; # ‐ for comparison of 48 h versus theoretic; ^ ‐ for comparison of 72 h versus theoretic.

*Abbreviations*: C:D, CAR T cells versus DAMI cells, at different culture ratio; DAMI, DAMI Luc2+ cells at different time points; ns, not significant.

However, after 48 and 72 h, the inhibitory effect was overcome by the fast DAMI Luc2 growing rate, indicating that our CAR T cells may be exhausted after the first 24 h of co‐culture, in our in vitro models.

### 3.4 LDH activity determination to evaluate membrane integrity after 24 h of co‐culture

After the first 24 h of co‐culture, the inhibition of DAMI Luc2+ cells growth was different depending on the co‐culture ratio. To confirm that CAR T cells bind to DAMI Luc2+ cells and initiate membrane damage, we decided to evaluate the release of lactate dehydrogenase (LDH) which is a cytosolic enzyme which is involved in the cell respiratory mechanism by converting pyruvate to lactate and generating energy, therefore its dysregulation may indicate changes in the metabolism, shifts in the aerobic/anaerobic glycolysis or in the cell death mechanism.^25^ For the in vitro experiments, there are two ways to measure the cell death; by flow cytometry when evaluating apoptosis and necrosis, and by evaluating the membrane integrity based on the LDH release. These two methods are also complementary to morphological evaluation of the nuclei and cellular compartments and the measurement of a time‐dependent toxicity by cell death markers.^26^ The apoptosis and necrosis assay by Annexin V‐FITC and PI staining is one of the study limitations, due to the GFP green fluorescent signal present in CAR T cells, we cannot perform the apoptosis/necrosis assay, the FITC from Annexin V‐FITC will overlap with GFP signal, generating false positive results.^27^, ^28^

A membrane integrity assay was performed by analysing the enzymatic activity of LDH that was released in the cell culture medium in each experimental group and we compared the release LDH from co‐cultured cells to the CAR T cells and DAMI Luc2+ cells cultured alone. The group with CAR T cells and DAMI Luc2 Ratio of 1:9 has the most intense LDH activity compared to DAMI Luc2+ with a *p* value of 0.0007, while at a ratio of 1:4 the *p* value was 0.0012. As we previously mentioned at the flow cytometry analysis, when we added CAR T cells at a ratio of 4:1 and 9:1, they created clusters and less CAR T cells were available to bind DAMI Luc2, with a *p* value of 0.0176 and 0.04. Only the 1:1 ratio showed no difference in the LDH activity compared to the control group.

**FIGURE 4:**
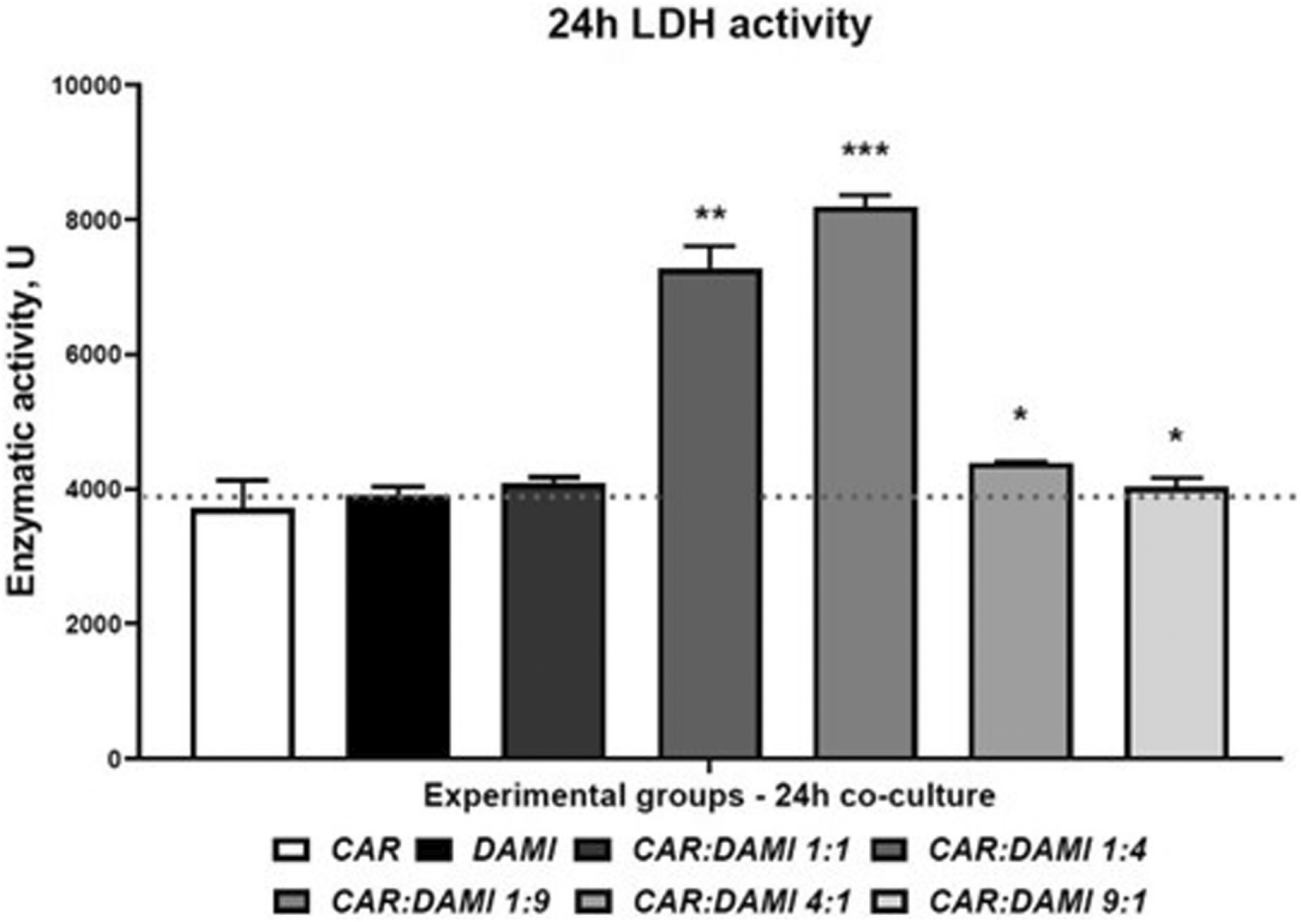
Lactate dehydrogenase (LDH) activity representation after 24 h of co‐culture between DAMI Luc2 cells and CAR T cells. the experimental groups were compared to DAMI group that was not co‐cultured with the CAR T cells, the results after 24 h of incubation were analysed using the two‐tailed paired t‐test, with *p* < 0.05*, *p* < 0.01** and *p* < 0.001***. For all the analysed groups, *n* = 3. Legend: CAR, CAR T cells; C:D, CAR versus DAMI cells, at different culture ratio; DAMI, DAMI Luc2+ cells; ns, not significant.

As presented in Figure 4 and Table 2, the groups that have less CAR T cells initially seeded present the highest LDH activity compared to the group containing DAMI Luc2+ alone. The evaluation at 48 and 72 h was not relevant due to the DAMI Luc2+ cells growth where the Flow cytometry analysis indicated that CAR T cells are no longer effective.

**TABLE 2:** The experimental groups were compared to DAMI group that was not co‐cultured with the CAR T cells, the results after 24 h of incubation were analysed using the two‐tailed paired *t*‐test, with *p* < 0.05*, *p* < 0.01** and *p* < 0.001***.

DAMI versus CAR	DAMI versus C:D 1:1	DAMI versus C:D 1:4	DAMI versus C:D 1:9	DAMI versus C:D 4:1	DAMI versus C:D 9:1
0.3908 ns	0.2699 ns	0.0012**	0.0007***	0.0176*	0.04*

*Note*: For all the analysed groups, *n* = 3.

*Abbreviations*: CAR, CAR T cells; C:D, CAR versus DAMI cells, at different culture ratio; DAMI, DAMI Luc2+ cells; ns, not significant.

### 3.5 IVIS evaluation of the luciferin signal

Target cells expressing luciferase signal were co‐cultured with CAR T cells and Mock at same ratio as for LDH assay. The evaluation was performed after 24 h of incubation. In the 96 well plate, the cells were seeded at a density of 10,000 cells per well in a final volume of 200 μL. After 1 day of incubation, 3 μL of RediJect D‐Luciferin were added to each well, including the controls and the wells that had only culture media. The cells were incubated for 10 min at 37°C and then the plate was evaluated by IVIS.

**FIGURE 5:**
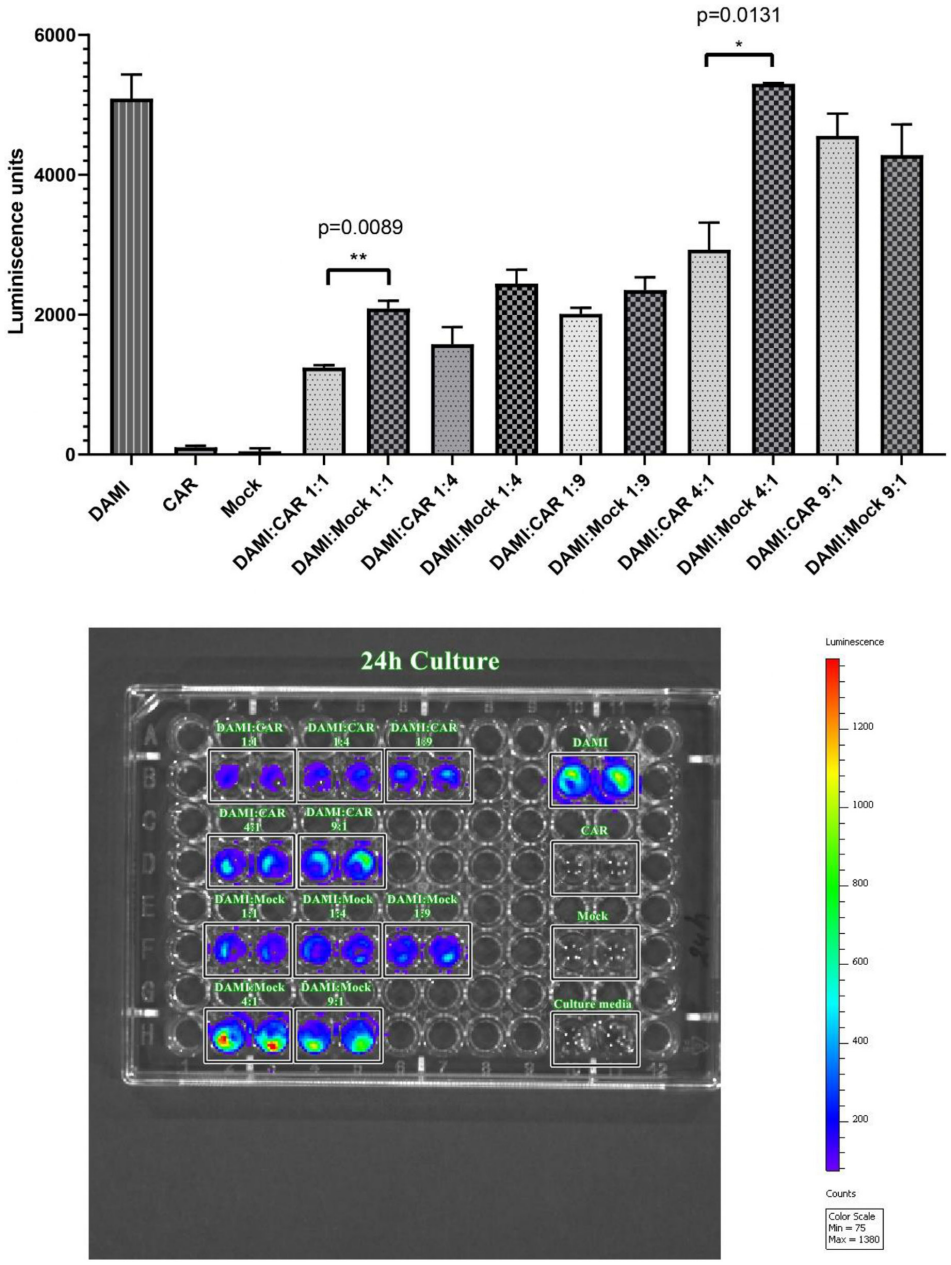
Luciferase signal evaluation after 24 h culture of DAMI cells with CAR T cells and Mock. The luminescent signal was evaluated after 24 h of incubation using the two‐tailed paired *t*‐test, with *p* < 0.05*.

As presented in Figure 5, after 24 h of incubation of different E:T ratio the most significant differences were observed between DAMI: CAR 1:1 and DAMI: Mock 1:1 with a *p* value of 0.0089 and between DAMI: CAR 4:1 and DAMI: Mock 4:1 with a p value of 0.0131. The inhibitory effect of CAR T cells on DAMI Luc2 cells is highlighted in the figure depicting the 96 well plate with the co‐culture groups. The luminescent signal is more intense when Mock is replacing CAR T cells. This effect suggests that the CAR T cells have an inhibitory effect on target cells with limited unspecific binding. The trend is visible in all experimental groups, excepting the group with nine Target cells to one effector.

The luminescent signal is not present in CAR T cells, mock cells and culture media, only the groups containing DAMI Luc2 cells were expressing luminescent signal.

### 3.6 TNF alpha release assay

The co‐culture groups were evaluated after 24 h using an ELISA Assay to determine the quantity of TNF alpha released in the culture media after the effector cells were incubated with target cells, respecting the same E:T ratios as for the other assays.

The TNF alpha released in the culture media was visibly higher in the groups with CAR T cells and lower in the groups where CAR T cells were replaced with mock. However, significant differences were observed between C:D 1:4 and M:D 1:4 with a *p* value of 0.0138 and between C:D 4:1 and M:D: 4:1 with a *p* value of 0.0469.

The release of TNF alpha in the groups with CAR T cells targeting DAMI Luc2 indicates that the binding of effectors to target cells trigger the release of specific cytokines which will stimulate the inhibition of M7‐AMkL cells.

### 3.7 Preclinical murine testing of CAR‐T cells against M7‐AML Luc2 positive cells

The NSG‐S female mice were conditioned with busulfan, and after 24 h 2 million DAMI Luc2 cells were injected in the Knee Joint to induce M7‐AML. After 20 days the mice were evaluated under IVIS system and as a pilot test we inoculated one mouse with 2 million CAR T cells, one mouse with 10 million CAR T cells, while one control mouse was injected with saline buffer without CAR T cells.

**FIGURE 6:**
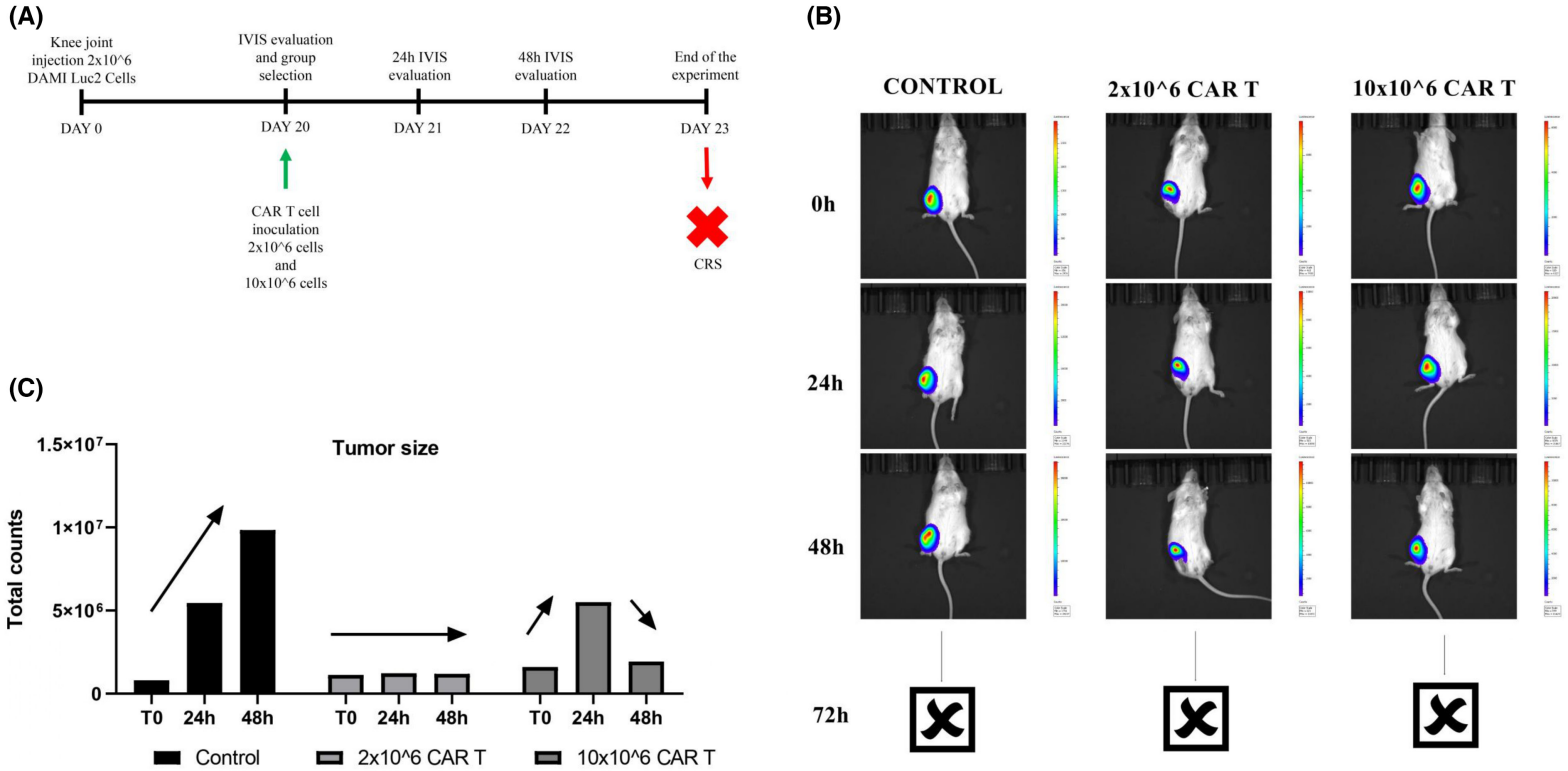
In vivo determination of the CAR T inhibitory effect on DAMI cells. The timeframe graphic (A) highlights the DAMI cells inoculation day, the day when CAR T cells were added and the periodical evaluation using IVIS. IVIS captures (B) at 0, 24 and 48 h show the tumour size based on its intensity, and the tumour size is represented using the total luminescence counts (C).

The IVIS signal is presented in Figure 6. The luminescent signal in control was increased in a time dependent manner, while in the group where 2 million CAR T cells were injected the signal was constant after 24 and 48 h. In the group where 10 million CAR T cells were injected, the luminescent signal is initially increased after 24 h, then the signal is decreased after 48 h. Unfortunately, the experiment was stopped after the 72 h when the animals injected with CAR T cells died.

Based on the in vitro results where the release of TNF alpha was increased when CAR T cells were co‐cultured with DAMI for 24 h, we believe that the death of the mice occurred due to a cytokine release syndrome.

## 4 DISCUSSION

With the immune system as a key player in the progression and metastasis of cancer cells, immune system cells are suitable to be used as therapeutic agents. CAR T cells are promising antitumor agents against B cell lineages. The treatment and response duration may vary from patient to patient, nonetheless side effect can occur, or some patients can later develop post‐therapy infections, while some are relapsed/refractory. Cytokine release syndrome (CRS) is one of the most common side effects result due to the CAR T cell therapy induced toxicity, however CRS and neurotoxicity can occur with different intensities depending on each case. With proper intensive care protocols and constant monitoring, patients have high chances to survive and increase the quality of life.^17^, ^29^, ^30^, ^31^, ^32^, ^33^, ^34^, ^35^

The most common CAR T cells target CD19, being the result of 10 years of research and innovation, thus, five CAR T cell therapies are now approved by the US Food and Drug Administration (FDA) and European Medicines Agency (EMA), targeting B‐cell lymphomas, multiple myeloma and acute B‐cell lymphoblastic leukaemia, while CAR T cells against solid tumours showed inconsistent results.^36^, ^37^, ^38^, ^39^, ^40^

As the in vitro testing is essential before translating the study to animal models, the co‐culture between CAR T cells and the target may indicate the potential efficacy of these CAR T cells. By lowering the ratio between effector and target, we can recreate the ‘real life’ situation when CAR T cells represent a minority in the organism after infusion,^41^ thus by analysing the interaction between CAR T cells and the target at different ratios, many situations can be evaluated.

After the evaluation of different co‐culture ratios, our data show that within the groups with lower ratio between effector and target (group C:D 1:4 and C:D 1:9) after 24 h of co‐culture DAMI Luc2 cell population was reduced compared to the initial seeding ratio, showing a high statistically significance with *p* values of 0.0097 and 0.0011. On the other hand, when the ratio was higher, and more CAR T cells were added compared to the target, was observed a high increase of DAMI Luc2 cell population with statistically significant *p* values of 0.0158 and 0.0023.

In vitro, our newly described CAR T cells, when seeded at a high density, tend to generate clusters, and increase their proliferation rate while maintaining the clusters. Furthermore, the clusters showed an increase in their size. We believe that the CAR T cells that are trapped in the clusters are less active and are not able to bind the target cells. Thus, our results show a better response when choosing a lower ratio between effector and target. However, after 48 and 72 h the target cells population increased, in all groups, without exception, and with high statistically significance after 72 h of co‐culture in C:D 4:1 and C:D 9:1 group, showing a *p* value of 0.0060 and 0.0025. In the case of lower ratio between effector and target cells, the DAMI Luc2 population reaches a plateau, and the cell density in the dish increases and consumed the nutrients that were available for cell growth. These results indicate that our new CAR T cells have limited effect on in vitro studies and need more stimuli to maintain their activation.

During the CAR T cell activation, the target cells suffer membrane damage, and until exhaustion CAR T cells stimulate the membrane damage of targets by releasing cytokines, granzyme and perforins in the extracellular matrix. For in vitro studies, the co‐culture between CAR T cells and the target showed some limitations, due to the lack of myeloid cell in co‐culture, the activation is limited to the soluble factors which stimulate the anti‐tumour response.^42^

To assess the cytotoxicity of our new CAR T cells, we performed a LDH release assay, and we tested only the 24 h co‐culture groups where we observed the decrease and the increase of the DAMI Luc2 cell population. LDH release indicates that the membrane is damaged, and the cytotoxicity is increased.^43^ Thus, we evaluated the LDH release in the culture media comparing the co‐cultured groups to the control representing DAMI Luc2 cells and CAR T cells alone in culture. The results showed a statistically significant increase in LDH release in the groups with a lower ratio between effector and target, with a *p* value of 0.0012 and 0.0007. On the other hand, the LDH release was lower in the case of a higher ratio between effector and target cells, with the *p* values that reached statistically significance value of 0.0176 and 0.04.

The increased LDH release was observed in the same groups where we observed a reduced population of DAMI Luc2 cells, indicating that the cellular integrity within these groups was affected.

For the evaluation of the unspecific effects of CAR T cells, both CAR T cells and Mock cells were evaluated by flow cytometry for 24 h. The experimental groups included different ratios of E:T ratios and all the experimental groups that had CAR T cells showed lower number and percentages of CD41 positive cells compared to the Mock groups where the percentages were almost similar to the theoretical value. The results are presented in Table S1. The inhibitory effect of CAR T cells over M7‐AMkL cells is promising and shows that CD41 can be used as a potential therapeutical target.

**FIGURE 7:**
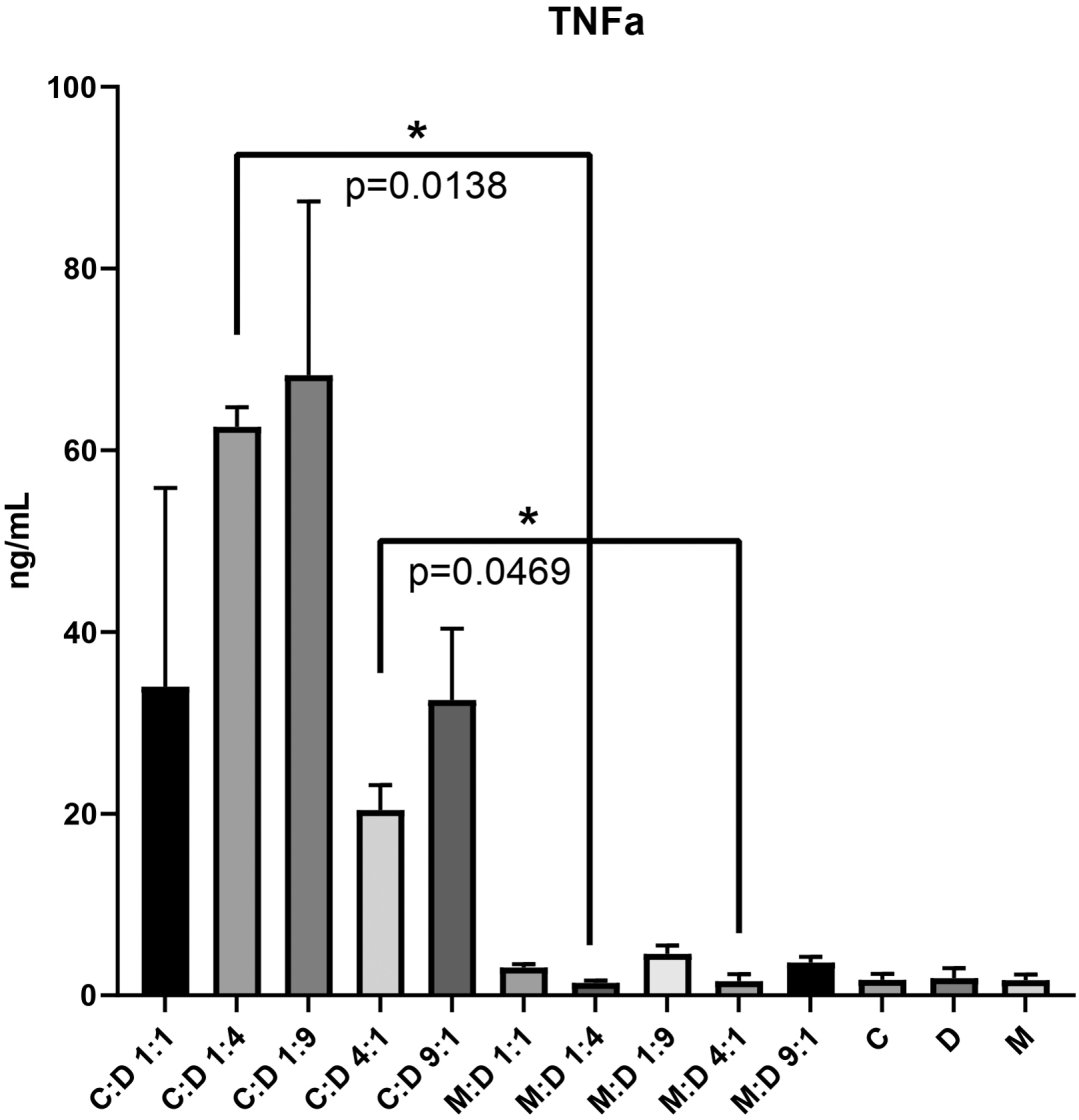
TNFα secreted after 24 h co‐culture of DAMI cells with CAR T cells and Mock. The results after 24 h of incubation were analysed using the two‐tailed paired *t*‐test, with *p* < 0.05*.

TNF alpha release (Figure 7) was also observed in the groups with CAR T cells and not in the groups with Mock. This is a sign of activation and proliferation of the CAR T cells in the presence of target cells. After CAR T cell infusion, cytokines such as INFƔ, TNF alpha, IL‐6 or IL‐10 become more elevated and can lead to several side effects associated to CRS.^44^, ^45^, ^46^

The inhibitory effect of the CAR T cells is confirmed by the luciferase evaluation by IVIS, where the Mock allowed target cells to proliferate and generate intense luminescent signal (Figure 5) while the luminescent signal was lower when Mock cells were replaced by CAR T cells at the same E:T ratios. The CD41 positive cells were significantly decreased in all experimental groups with CAR T cells compared to Mock, the results being correlated with the TNF alpha release and luminescent evaluation assay, indicating that the herein tested CAR T cells can target CD41 and can inhibit the M7‐AMkL cells proliferation.

The in vitro results are confirmed by the in vivo evaluations presented in Figure 6. After 48 h the tumour luminescent signal was decreased or maintained as at the beginning of the CAR T cells injection. The release of TNF alpha in all experimental groups containing CAR T cells can be related to the intense cytotoxic effect after CAR T cells bind to the target, thus the animal death that occurred after 72 h of exposure to CAR T cells can be explained by a potential CRS. The side effects, such as neurotoxicity or CRS are the major issues in CAR T cell therapies, and other research groups have reported CRS after exposure to CAR T cells which have other targets in M7‐AMkL, such as folate receptor alpha (FOLR1).^47^

This preliminary study is the first in Romania describing a newly designed CAR T cell construct and afterwards tested in the preclinical setting; and it highlighted the limited effect of our CAR T cells which target CD41 protein on AMkL malignant cell surface. Improvements are thus needed for our CAR T cells to target with higher efficacy the CD41 marker and to reduce the side effects resulted after in vivo CAR T cell treatment.

## 5 CONCLUSION

The anti‐CD‐41 CAR T cells showed good response for 24 h interaction with M7‐AML cells when evaluating the in vitro experimental groups and had limited effect after 48 and 72 h of interaction, when tumour cells increased their population. The cytotoxicity that was induced after 24 h, was observed mostly in the groups with lower E:T ratio. We have two options for the future, to generate bispecific CAR T cells which can target CD41 and other specific receptor for M7‐AML and the second option is to test other ratios between effector and target cells.

## AUTHOR CONTRIBUTIONS

**Adrian‐Bogdan Tigu:** Formal analysis (equal); investigation (equal). **Catalin Sorin Constantinescu:** Data curation (equal); formal analysis (equal). **Patric Teodorescu:** Investigation (equal). **David Kegyes:** Investigation (equal). **Raluca Munteanu:** Investigation (equal). **Richard Feder:** Investigation (equal). **Mareike Peters:** Investigation (equal). **Ioana Pralea:** Investigation (equal). **Cristina Iuga:** Investigation (equal). **Diana Cenariu:** Investigation (equal). **Andra Marcu:** Investigation (equal). **Alina Tanase:** Investigation (equal). **Anca Colita:** Investigation (equal). **Rares Drula:** Investigation (equal). **Jon Thor Bergthorsson:** Investigation (equal). **Victor Greiff:** Conceptualization (equal). **Delia Dima:** Investigation (equal). **Cristina Selicean:** Formal analysis (equal). **Ioana Rus:** Investigation (equal). **Mihnea Zdrenghea:** Formal analysis (equal). **Diana Gulei:** Investigation (equal). **Gabriel Ghiaur:** Funding acquisition (equal); project administration (equal). **Ciprian Tomuleasa:** Conceptualization (equal); project administration (equal).

## CONFLICT OF INTEREST STATEMENT

No potential conflict of interest is reported.

## Supporting information


Figure S1.

Table S1.

Table S2.

Table S3.

Table S4.

## Data Availability

The data that support the findings of the study are available from the corresponding author upon reasonable request.
