# Supplementary material for: Design and preclinical testing of an anti‐CD41 CAR T cell for the treatment of acute megakaryoblastic leukaemia
Source: J Cell Mol Med. 2023 Sep 4;27(19):2864–75. doi: 10.1111/jcmm.17810 (PMC10538266; doi:10.1111/jcmm.17810)
Supplement: Supplementary file 1 — Figure S1. [file JCMM-27-2864-s005.docx]

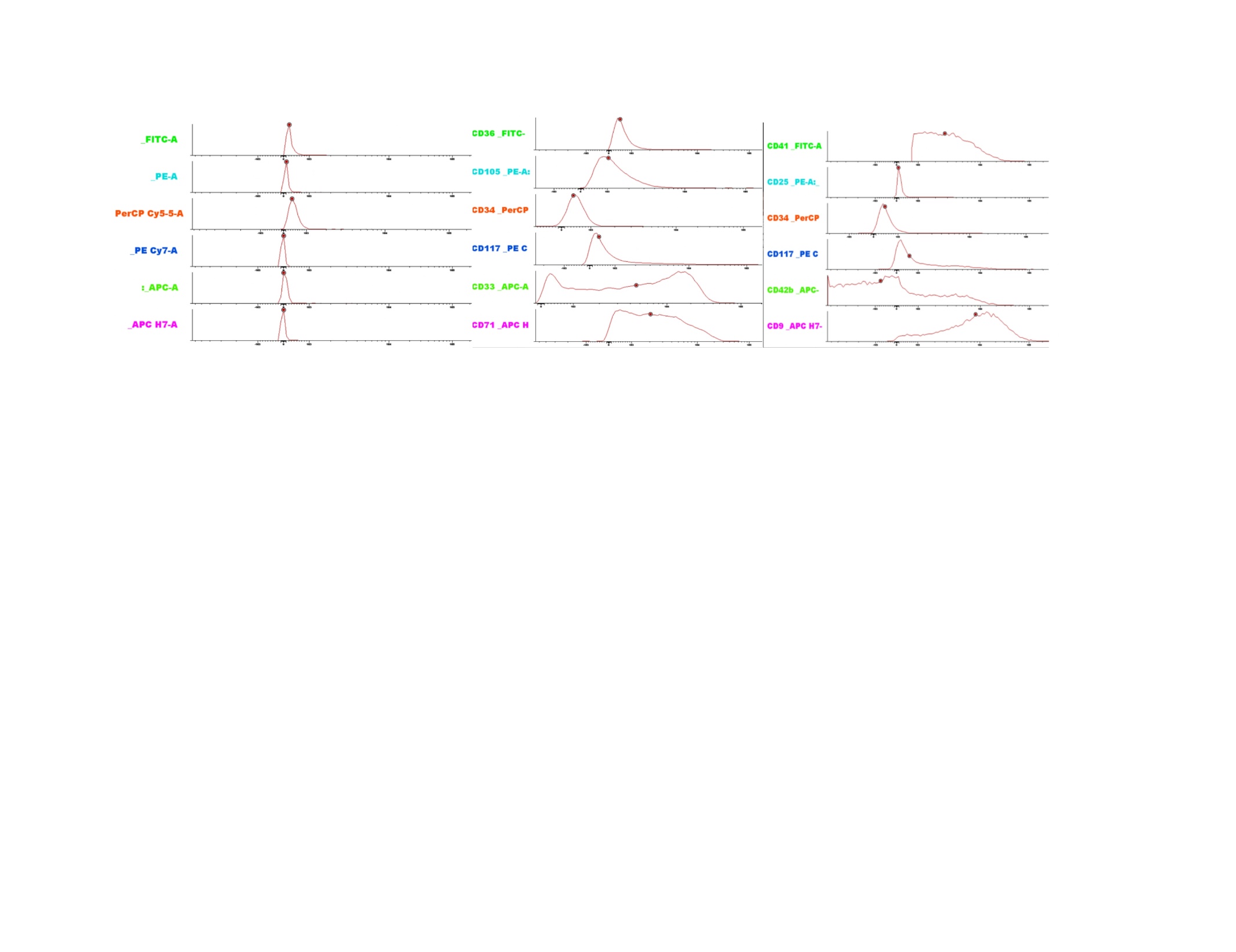


**Supplementary File A**

**Supplemental Figure -** **The evaluation of DAMI Luc2 cells using Euroflow consortium recommendation for megakaryocytic and erythroid markers.** Based on the Euroflow panel used we concluded that our DAMI Luc2 cell line can be included in the megakaryocytic group, thus the erythroid markers specific for M6 AML were not expressed. The analyzed cells express megakaryocytic markers CD41 and CD9. The cells were analyzed using megakaryocytic and erythroid markers recommended by the Euro Flow consortium. The analyzed cells express megakaryocytic markers CD41 and CD9. CD42 expression is heterogenous. The dim heterogenous expression of CD71 is in our experience with this clone unspecific, as it can often been seen in immature cells of various lineages, while true erythroid cells express CD71 at a high level.
