# Supplementary material for: Design and preclinical testing of an anti‐CD41 CAR T cell for the treatment of acute megakaryoblastic leukaemia
Source: J Cell Mol Med. 2023 Sep 4;27(19):2864–75. doi: 10.1111/jcmm.17810 (PMC10538266; doi:10.1111/jcmm.17810)
Supplement: Supplementary file 2 — Table S1. [file JCMM-27-2864-s003.docx]

**Supplementary File B**

**Supplemental table - CD41+ cells in DAMI:CAR and DAMI:MOCK coculture after 24h**

| **Experimental group** | **CD41 Theoretical %** | **CD41+ cells (%)** | **CD41+ cells** | **Total alive cells** |
| --- | --- | --- | --- | --- |
| **DAMI Luc2** | 100% | 95.3% | 9671 **~** | 9974 |
| **DAMI Luc2** | 100% | 97.6% | 9035 **~** | 9260 |
|  |  |  |  |  |
| **DAMI:CAR 1:1** | 50% | 36% | 3451 **↓** | 9580 |
| **DAMI:CAR 1:1** | 50% | 34.5% | 3325 **↓** | 9625 |
| **DAMI:MOCK 1:1** | 50% | 48% | 4512 **~** | 9401 |
| **DAMI:MOCK 1:1** | 50% | 47.6% | 4531 **~** | 9510 |
|  |  |  |  |  |
| **DAMI:CAR 4:1** | 80% | 65.9% | 6221 **↓** | 9439 |
| **DAMI:CAR 4:1** | 80% | 66% | 6225 **↓** | 9434 |
| **DAMI:MOCK 4:1** | 80% | 76.4% | 7152 **~** | 9362 |
| **DAMI:MOCK 4:1** | 80% | 76.2% | 7149 **~** | 9376 |
|  |  |  |  |  |
| **DAMI:CAR 1:4** | 20% | 12.9% | 1267 **↓** | 9845 |
| **DAMI:CAR 1:4** | 20% | 12.5% | 1234 **↓** | 9836 |
| **DAMI:MOCK 1:4** | 20% | 17.3% | 1659 **~** | 9568 |
| **DAMI:MOCK 1:4** | 20% | 17.8% | 1702 **~** | 9571 |
| Legend: Comparison between CAR and MOCK effect is highlighted with an arrow at the CAR: DAMI groups. In all three co-culture conditions, after 24h, DAMI cells (CD41+) were inhibited by CAR T cells. **“~”** indicates the CD41+ similar cell number compared to the theoretical value; **“↓”** indicates the decreased CD41+ cell number compared to the theoretical value. | | | | |
