# Supplementary material for: Design and preclinical testing of an anti‐CD41 CAR T cell for the treatment of acute megakaryoblastic leukaemia
Source: J Cell Mol Med. 2023 Sep 4;27(19):2864–75. doi: 10.1111/jcmm.17810 (PMC10538266; doi:10.1111/jcmm.17810)
Supplement: Supplementary file 3 — Table S2. [file JCMM-27-2864-s002.docx]

**Supplementary Table 1. ScFv sequence**

**scFv**

EIVLTQSPVTLSVTPGDSVSLSCRASRDISNNLHWFQQTSHESPRLLIKYASQSMSGIPSRFSGSGSGTDFTLSINSVETEDFGMYFCQQTNSWPYTFGGGTKLEIKGGGGSGGGGSGGGGSEVQLQQSGTVLARPGASVKMSCEASGYTFTNYWMHWVKQRPGQGLEWIGAIYPGNSDTSYIQKFKGKAKLTAVTSTTSVYMELSSLTNEDSAVYYCTLYDGYYVFAYWGQGTLVTVSA
