# Supplementary material for: Design and preclinical testing of an anti‐CD41 CAR T cell for the treatment of acute megakaryoblastic leukaemia
Source: J Cell Mol Med. 2023 Sep 4;27(19):2864–75. doi: 10.1111/jcmm.17810 (PMC10538266; doi:10.1111/jcmm.17810)
Supplement: Supplementary file 4 — Table S3. [file JCMM-27-2864-s001.docx]

**Supplementary Table 2. Nucleotide sequence of the CAR cassette**

**EF-1alpha promoter-Signal peptide-scFv-CD8 hinge-CD28 TM and ICD-4-1BB-CD3ζ-T2A-GFP**

GAGTAATTCATACAAAAGGACTCGCCCCTGCCTTGGGGAATCCCAGGGACCGTCGTTAAACTCCCACTAACGTAGAACCCAGAGATCGCTGCGTTCCCGCCCCCTCACCCGCCCGCTCTCGTCATCACTGAGGTGGAGAAGAGCATGCGTGAGGCTCCGGTGCCCGTCAGTGGGCAGAGCGCACATCGCCCACAGTCCCCGAGAAGTTGGGGGGAGGGGTCGGCAATTGAACCGGTGCCTAGAGAAGGTGGCGCGGGGTAAACTGGGAAAGTGATGTCGTGTACTGGCTCCGCCTTTTTCCCGAGGGTGGGGGAGAACCGTATATAAGTGCAGTAGTCGCCGTGAACGTTCTTTTTCGCAACGGGTTTGCCGCCAGAACACAGGTAAGTGCCGTGTGTGGTTCCCGCGGGCCTGGCCTCTTTACGGGTTATGGCCCTTGCGTGCCTTGAATTACTTCCACGCCCCTGGCTGCAGTACGTGATTCTTGATCCCGAGCTTCGGGTTGGAAGTGGGTGGGAGAGTTCGAGGCCTTGCGCTTAAGGAGCCCCTTCGCCTCGTGCTTGAGTTGAGGCCTGGCTTGGGCGCTGGGGCCGCCGCGTGCGAATCTGGTGGCACCTTCGCGCCTGTCTCGCTGCTTTCGATAAGTCTCTAGCCATTTAAAATTTTTGATGACCTGCTGCGACGCTTTTTTTCTGGCAAGATAGTCTTGTAAATGCGGGCCAAGATCTGCACACTGGTATTTCGGTTTTTGGGGCCGCGGGCGGCGACGGGGCCCGTGCGTCCCAGCGCACATGTTCGGCGAGGCGGGGCCTGCGAGCGCGGCCACCGAGAATCGGACGGGGGTAGTCTCAAGCTGGCCGGCCTGCTCTGGTGCCTGGCCTCGCGCCGCCGTGTATCGCCCCGCCCTGGGCGGCAAGGCTGGCCCGGTCGGCACCAGTTGCGTGAGCGGAAAGATGGCCGCTTCCCGGCCCTGCTGCAGGGAGCTCAAAATGGAGGACGCGGCGCTCGGGAGAGCGGGCGGGTGAGTCACCCACACAAAGGAAAAGGGCCTTTCCGTCCTCAGCCGTCGCTTCATGTGACTCCACGGAGTACCGGGCGCCGTCCAGGCACCTCGATTAGTTCTCGAGCTTTTGGAGTACGTCGTCTTTAGGTTGGGGGGAGGGGTTTTATGCGATGGAGTTTCCCCACACTGAGTGGGTGGAGACTGAAGTTAGGCCAGCTTGGCACTTGATGTAATTCTCCTTGGAATTTGCCCTTTTTGAGTTTGGATCTTGGTTCATTCTCAAGCCTCAGACAGTGGTTCAAAGTTTTTTTCTTCCATTTCAGGTGTCGTGATTCGAATTCGCCACCATGGCCTTACCAGTGACCGCCTTGCTCCTGCCGCTGGCCTTGCTGCTCCACGCCGCCAGGCCGCGCCACCATGGCCTTACCAGTGACCGCCTTGCTCCTGCCGCTGGCCTTGCTGCTCCACGCCGCCAGGCCGGAGATCGTGCTGACCCAGTCCCCCGTGACACTGAGCGTGACACCTGGCGATTCCGTGTCCCTGTCCTGCAGGGCCAGCAGGGATATCAGCAACAACCTGCACTGGTTTCAGCAGACAAGCCACGAGTCCCCCAGACTGCTGATCAAGTACGCCTCCCAGTCCATGTCCGGCATCCCTTCCAGGTTCTCCGGCTCCGGCAGCGGCACAGACTTTACACTGTCCATCAATTCCGTGGAGACCGAGGACTTCGGCATGTACTTCTGCCAGCAGACCAACTCCTGGCCCTACACATTTGGCGGCGGCACCAAGCTGGAGATCAAGGGCGGCGGCGGCTCCGGAGGAGGAGGAAGCGGAGGAGGCGGCAGCGAGGTGCAGCTGCAGCAGAGCGGCACCGTGCTGGCCAGGCCTGGAGCTTCCGTGAAGATGAGCTGCGAGGCCTCCGGCTACACATTCACCAATTACTGGATGCACTGGGTGAAGCAGAGACCCGGCCAGGGCCTGGAGTGGATCGGCGCTATCTACCCTGGCAACTCCGACACCTCCTACATCCAGAAGTTCAAGGGCAAGGCCAAGCTGACCGCCGTGACATCCACAACCTCCGTGTACATGGAGCTGTCCAGCCTGACAAACGAGGATTCCGCCGTGTACTACTGTACCCTGTACGATGGCTACTACGTGTTCGCCTACTGGGGCCAGGGCACACTGGTGACCGTGAGCGCCACCACTACCCCAGCACCGAGGCCACCCACCCCGGCTCCTACCATCGCCTCCCAGCCTCTGTCCCTGCGTCCGGAGGCATGTAGACCCGCAGCTGGTGGGGCCGTGCATACCCGGGGTCTTGACTTCGCCTGCGATGTAGACCCGCAGCTGGTGGGGCCGTGCATACCCGGGGTCTTGACTTCGCCTGCGATTTCTGGGTGCTGGTCGTTGTGGGCGGCGTGCTGGCCTGCTACAGCCTGCTGGTGACAGTGGCCTTCATCATCTTTTGGGTGAGGAGCAAGCGGAGCAGACTGCTGCACAGCGACTACATGAACATGACCCCCCGGAGGCCTGGCCCCACCCGGAAGCACTACCAGCCCTACGCCCCTCCCAGGGATTTCGCCGCCTACCGGAGCGGGATTTCGCCGCCTACCGGAGCAAGCGCGGTCGGAAGAAGCTGCTGTACATCTTTAAGCAACCCTTCATGAGGCCTGTGCAGACTACTCAAGAGGAGGACGGCTGTTCATGCCGGTTCCCAGAGGAGGAGGAAGGCGGCTGCGAACTGGCAGACTACTCAAGAGGAGGACGGCTGTTCATGCCGGTTCCCAGAGGAGGAGGAAGGCGGCTGCGAACTGCGCGTGAAATTCAGCCGCAGCGCAGATGCTCCAGCCTACAAGCAGGGGCAGAACCAGCTCTACAACGAACTCAATCTTGGTCGGAGAGAGGAGTACGACGTGCTGGACAAGCGGAGAGGACGGGACCCAGAAATGGGCGGGAAGCCGCGCAGAAAGAATCCCCAAGAGGGCCTGTACAACGAGCTCCAAAAGGATAAGATGGCAGAAGCCTATAGCGAGATTGGTATGAAAGGGGAACGCAGAAGAGGCAAAGGCCACGACGGACTGTACCAGGGACTCAGCACCGCCACCAAGGACACCTATGACGCTCTTCACATGCAGGCCCTGCCGCCTCGGGAGGGCAGAGGCAGCCTGCTGACATGTGGCGACGTGGAAGAGAACCCTGGCCCCTGCCGCCTCGGGAGGGCAGAGGCAGCCTGCTGACATGTGGCGACGTGGAAGAGAACCCTGGCCCCATGGTGAGCAAGGGCGAGGAGCTGTTCACCGGGGTGGTGCCCATCCTGGTCGAGCTGGACGGCGACGTAAACGGCCACAAGTTCAGCGTGTCCGGCGAGGGCGAGGGCGATGCCACCTACGGCAAGCTGACCCTGAAGTTCATCTGCACCACCGGCAAGCTGCCCGTGCCCTGGCCCACCCTCGTGACCACCCTGACCTACGGCGTGCAGTGCTTCAGCCGCTACCCCGACCACATGAAGCAGCACGACTTCTTCAAGTCCGCCATGCCCGAAGGCTACGTCCAGGAGCGCACCATCTTCTTCAAGGACGACGGCAACTACAAGACCCGCGCCGAGGTGAAGTTCGAGGGCGACACCCTGGTGAACCGCATCGAGCTGAAGGGCATCGACTTCAAGGAGGACGGCAACATCCTGGGGCACAAGCTGGAGTACAACTACAACAGCCACAACGTCTATATCATGGCCGACAAGCAGAAGAACGGCATCAAGGTGAACTTCAAGATCCGCCACAACATCGAGGACGGCAGCGTGCAGCTCGCCGACCACTACCAGCAGAACACCCCCATCGGCGACGGCCCCGTGCTGCTGCCCGACAACCACTACCTGAGCACCCAGTCCGCCCTGAGCAAAGACCCCAACGAGAAGCGCGATCACATGGTCCTGCTGGAGTTCGTGACCGCCGCCGGGATCACTCTCGGCATGGACGAGCTGTACAAG
