# Supplementary material for: Design and preclinical testing of an anti‐CD41 CAR T cell for the treatment of acute megakaryoblastic leukaemia
Source: J Cell Mol Med. 2023 Sep 4;27(19):2864–75. doi: 10.1111/jcmm.17810 (PMC10538266; doi:10.1111/jcmm.17810)
Supplement: Supplementary file 5 — Table S4. [file JCMM-27-2864-s004.docx]

**Supplementary Table 3. Aminoacid sequence of the CAR cassette.**

**Signal peptide-scFv-CD8 hinge-CD28 TM and ICD-4-1BB-CD3ζ-T2A-iCas9**

MALPVTALLLPLALLLHAARPEIVLTQSPVTLSVTPGDSVSLSCRASRDISNNLHWFQQTSHESPRLLIKYASQSMSGIPSRFSGSGSGTDFTLSINSVETEDFGMYFCQQTNSWPYTFGGGTKLEIKGGGGSGGGGSGGGGSEVQLQQSGTVLARPGASVKMSCEASGYTFTNYWMHWVKQRPGQGLEWIGAIYPGNSDTSYIQKFKGKAKLTAVTSTTSVYMELSSLTNEDSAVYYCTLYDGYYVFAYWGQGTLVTVSATTTPAPRPPTPAPTIASQPLSLRPEACRPAAGGAVHTRGLDFACDFWVLVVVGGVLACYSLLVTVAFIIFWVRSKRSRLLHSDYMNMTPRRPGPTRKHYQPYAPPRDFAAYRSKRGRKKLLYIFKQPFMRPVQTTQEEDGCSCRFPEEEEGGCELRVKFSRSADAPAYKQGQNQLYNELNLGRREEYDVLDKRRGRDPEMGGKPRRKNPQEGLYNELQKDKMAEAYSEIGMKGERRRGKGHDGLYQGLSTATKDTYDALHMQALPPREGRGSLLTCGDVEENPGPMVSKGEELFTGVVPILVELDGDVNGHKFSVSGEGEGDATYGKLTLKFICTTGKLPVPWPTLVTTLTYGVQCFSRYPDHMKQHDFFKSAMPEGYVQERTIFFKDDGNYKTRAEVKFEGDTLVNRIELKGIDFKEDGNILGHKLEYNYNSHNVYIMADKQKNGIKVNFKIRHNIEDGSVQLADHYQQNTPIGDGPVLLPDNHYLSTQSALSKDPNEKRDHMVLLEFVTAAGITLGMDELYK
